# Histological changes in the oral mucosa of the wistar rat treated with commercial lime (calcium hydroxide)--an optical and submicroscopic study.

**DOI:** 10.1038/bjc.1968.38

**Published:** 1968-06

**Authors:** S. M. Sirsat, S. V. Kandarkar

## Abstract

**Images:**


					
303

HISTOLOGICAL CHANGES IN THE ORAL MUCOSA OF THE

WISTAR RAT TREATED WITH COMMERCIAL LIME (CALCIUM
HYDROXIDE)-AN OPTICAL AND SUBMICROSCOPIC STUDY

SATYAVATI M. SIRSAT AND S. V. KANDARKAR

From the Cancer Research Institute, Parel, Bombay-12, India

Received for publication, December 12, 1967

THE high incidence of oral cancer in different population groups in many
south-east Asian nations has been stressed repeatedly in early and recent reports
(Orr, 1933; Balendra, 1949; Cooray, 1944; Khanolkar, 1959; Muir, 1962; Pind-
borg, 1965). In India, and elsewhere, epidemiological and biological investiga-
tions have been done, or are in progress, on any possible correlation between the
habitual use of the betel chew or " pan " and oral cancer. The involvement of
the main constituents of " pan "-betel leaf (Piper betel), betel nut (Areca catechu),
tobacco (Nicotiana tabacum), slaked shell or stone lime (slaked calcium hydroxide)
and catechu (Acacia catechu) have also been considered individually or in combina-
tion (Mody and Ranadive, 1959; Muir and Kirk, 1960; Dunham, Muir and Ham-
ner, 1966). The role of lime appears rather controversial, though some reports
do suggest its correlation with the occurrence of oral cancer. It was therefore
considered worthwhile to study the effects of slaked commercial lime on the oral
mucosa of laboratory animals. The main aims of the study were to evaluate the
part played by lime in the causation of oral malignancy or of oral conditions
considered to be precancerous and the trauma it caused to epithelial and sub-
epithelial tissue. This paper reports the histological changes produced on short
term and prolonged application of lime to the oral mucosa of normal, diet con-
ditioned and hormone treated Wistar rats.

MATERIAL AND METHODS

One hundred and thirty-nine weanling Wistar rats (66 male and 73 female)
were divided into four experimental groups as follows:

Group I-36 animals on standard laboratory diet (wheat 66%; cracked lentils
15%; pea nut 5 %; fish meal 7 %, shark liver oil 1 %, sesame oil 1 %).

Group 11-35 animals on a protein deficient diet (milled rice 1000 g.; NaCl 10 g.;
vegetable oil 50 ml.; cod liver oil 20 ml.; B-complex tablets (100 mg.) 3 tablets).

Group llI-36 animals on a vitamin B deficient diet (Sucrose 34.5%; corn
starch 34.0%; vegetable oil (vitamin A + D - 700 Units) 6-7%; vitamin free
casein 20%; NaCl 1.0%; CaCO3 0.5%).

Group IV-32 animals on standard laboratory diet, pretreated locally with
deoxycorticosterone acetate (DOCA). The hormone (Decortone-Cipla Ltd.,
Bombay) was injected on the palate biweekly in a dose of 1 mg. per injection.

SATYAVATI M. SIRSAT AND S. V. KANDARKAR

The experiment was started after pretreatment with 5 mg. in each animal. The
total hormone dosage was as follows:

Period of time             Total dosage

(months)   Total animals   (mg.)

1      .     6      .     12
2      .     6      .     21
3      .     5      .     29
4      .     6      .     38
5      .     5      .     47
6      .     4      .     56

A week after the introduction of solid diet, and in Group IV, after 5 mg. of
DOCA had been injected locally, the animals in each experiment were divided into
six groups, each consisting of one littermate control and five experimental animals.
The palate and buccal mucosa of all the experimental animals were painted daily,
for five days a week, with the test substance. The control animals were stroked
identically with a camel hair brush, simulating the mechanical irritation produced
during the oral painting. Animals in Group I were killed in batches of 6 (1
control and 5 experimental) at 2, 4, 6, 8, 10, and 12 months after start of painting.
Animals in the diet deficient groups were killed in batches of 6 (1 control and 5
experimental) at 2, 3, 4, 5, 6 and 7 months after start of painting, as it was difficult
to keep them alive for longer time periods. The hormone treated animals were
killed in 6 batches (1 control and 5 experimental) at 1, 2, 3, 4, 5 and 6 months after
start of painting.

The palate, tongue and buccal mucosa were fixed in Lillie's 10% neutral
formalin and divided in two portions for optical and electron microscopy.

6 ,u thick serial paraffin sections were stained with haematoxylin and eosin,
Mallory's trichrome, phosphotungstic acid haematoxylin (PTAH), Weigert's
resorcin fuchsin for elastica, aqueous 0.5% toluidine blue at pH 4-5.

For electron microscopy small pieces of palatal and buccal mucosal sub-
epithelial connective tissue were isolated under microscopic control from the
formalin fixed tissue, and washed for two hours in distilled water to remove traces
of the fixative. This material was then teased into a fine suspension of isolated
fibrils. Micro drops of this suspension were placed on coated 200 mesh copper
specimen grids. The samples were air dried, metal shadowed lightly at an angle
of 15-20? with chromium, and scanned in an RCA-EMU 2D electron microscope
with an objective aperture of 0.001" internal diameter.

The design of experiments in all four groups is shown in Table I.

The test substance used was slaked lime (calcium hydroxide) sold commer-
cially as a paste for use in the betel chew. Lime is prepared either from the
calcareous coating of marine invertebrates, harvested along the coast line, or
from stone quarried in three Indian provinces, Marwar, Uttar Pradesh or Bihar.
The chemical reaction involved in the commercial manufacture of this substance
is CaCO3 heat CaO + C02; CaO + H20 = Ca(OH)2.

RESULTS

In all four groups gross and microscopic observations on male and female
animals will be described together as no significant sex difference was observed in
the tissue response.

304

ORAL MUCOSA CHANGES IN THE WISTAR RAT

Macroscopic observations.-All control and experimental animals were weighed
weekly. Those on stock diet in Groups I and TV recorded a normal weight curve,
whereas animals maintained on a protein and vitamin B deficient diet in Groups
II and III showed gradual inanition and appeared generally unhealthy, with dull
hair and soreness of skin.

At the time of killing the majority of the animals in all four groups showed a
marked mauve discolouration of the oral mucosa.

Microscopic observations.-Table II gives a summary of the epithelial response
in control and experimental animals in all four groups.

A mild to moderate palatal hyperplasia occurred in 21 out of 24, and buccal
mucosal hyperplasia in 17 out of 24 control animals (Fig. 1 and 2). Other more
abnormal palatal epithelial changes were cell vacuolation in 7 animals, a promi-
nent stratum granulosum in 9 animals, hyperkeratosis in 5 animals and para-
keratosis in 1 animal. In the buccal mucosa these changes occurred as follows:
cell vacuolation in 6 animals, a prominent stratum granulosum in 9 animals,
hyperkeratosis in 5 animals and parakeratosis in 1 animal. No control animal
in any four groups showed cell atypia or acanthosis of the epithelium.

In a total of 115 palates painted with the lime the epithelial changes were mild
to moderate to massive hyperplasia and a prominent stratum granulosum in all
animals (Fig. 3 and 4), hyperkeratosis in 96 animals, marked cytoplasmic vacuo-
lation with a large nucleus and single or multiple nucleoli in 50 animals (Fig. 5
and 6). Attempts at invagination of rete pegs into the papillary layer were seen
in 59 animals (Fig. 3 and 4), whilst frank acanthosis was found in 26 animals
(Fig. 5 and 6). Similarly, in a total of 115 buccal mucosa painted with the
lime the changes were mild to moderate to massive hyperplasia and a prominent
stratum granulosum in all animals (Fig. 7 and 8) marked cytoplasmic vacuolation
with large nuclei in 71 animals, mild hyperkeratosis in 82 animals (Fig. 9, 10 and
11), invagination of rete pegs into the papillary layer in 59 animals and frank
acanthosis with cell atypia in 28 animals (Fig. 9, 10 and 11).

Table III summarises the connective tissue response in all the four groups. All
control and test animals in Groups II and Ill showed a scarcity of fibrous con-
nective tissue. In the 24 control animals, submucosal hyalinisation was seen in
12 animals, while fibroblastic proliferation was seen in animals kept on a B-
complex deficient diet and in all animals given the hormone. A mild inflam-
matory exudate was seen in 4 animals, 3 of whom belonged to Group IV where
injection injury had occurred. There was no dilatation and congestion of blood
vessels in any control animal. A progressively enhanced submucosal connective
tissue reaction was found in all test animals. In all four groups there was con-
siderable increase in fibroblastic proliferation (Fig. 6 and 11), oedema (Fig. 11),
connective tissue hyalinisation (Fig. 4 and 5), and a chronic inflammatory exudate.
Blood vessels were dilated and congested in 104 lime-exposed animals.

Fibroblasts in the protein and vitamin B deficient group had a foamy vacuo-
lated sparse cytoplasm, while those in the group of rats treated with DOCA were
large with abundant cytoplasm.

Three differential stains for connective tissue were used on serial sections in
this study. Table IV summarises the reaction of oral mucosal collagen to Mal-
lory's trichrome and phosphotungstic acid haematoxylin, (PTAH) and Weigert's
resorcin fuchsin for elastica. In the 24 control animals, the palatal collagen of
only one and the buccal mucosal collagen of only 2 animals showed tinctorial

305

SATYAVATI M. SIRSAT AND S. V. KANDARKAR

abnormality in occasional areas, identical coarse collagen staining pink with the
trichrome stain and purple with the PTAH. There was no increase in the resorcin
positive element in any tissue. In the 115 experimental animals, palatal collagen
of 49 animals and buccal mucosal collagen of 99 animals showed this tinctorial
alteration, in an occasional fibre bundle (Fig. 12 and 13).

The mast cell response, seen in Tables V and VI differed markedly in the
palate and the buccal mucosa. Mast cells were very few in the palate and few to
moderate in the buccal mucosa of all untreated control animals in the four groups.

EXPLANATION OF PLATES

FIG. 1 to 15 illustrate the tissue reaction in the oral mucosa of Wistar rats, irritated with a dry

camel hair brush or painted with commercial lime.

FIG. 1.-Section of the palate of diet control animal kept on a protein deficiency for 3 months.

A very mild epithelial hyperplasia and sparse connective tissue are seen. H. & E. x 65.
FIG. 2.-Section of the buccal mucosa of a control animal irritated with a dry camel hair

brush for 6 months. There is very mild epithelial hyperplasia and a prominent stratum
granulosum. No abnormal keratinisation or hyalinisation of the subepithelial connective
tissue is seen. H. & E. x 65.

FIG. 3.-Section of the palate from an animal painted for 4 months. There is moderate to

marked epithelial hyperplasia and a prominent stratum granulosum. Epithelial cords
invaginate into the corium. H. & E. x 78.

FIG. 4.-Section of the palate painted for 5 months. The epithelium is moderately to markedly

hyperplastic and has a very prominent stratum granulosum. Acanthosis of the epithelium
into the corium is seen. Subepithelium connective tissue is hyalinised. H. & E.  x 65.
FIG. 5. Section of the palate painted for 8 months showing marked hyperplasia, a prominent

stratum granulosum, and much acanthotic invagination into the corium. The subepithelial
connective tissue is sparse and hyalinised. H. & E. x 78.

FIG. 6.-Section of the palate from protein deficient animal painted for 6 months. There is

marked hyperplasia, a prominent stratum granulosum, nuclear vacuolation and acanthotic
invagination. Subepithelial areas show marked fibroblastic proliferation and inflammation.
H. &E.   x107.

FIG. 7.-Section of the palate from a protein deficient animal painted for 6 months. Epi-

thelial hyperplasia and a very prominent stratum granulosum are seen. Cell dissociation
and atypical staining of nucleoplasm are evident. H. & E. x 214.

FIG. 8. Section of the palate from a protein deficient animal painted for 6 months. Picture

shows an area of marked hyperplasia, with a prominent stratum granulosum and parakera-
tosis. Cell dissociation and vacuolation were also seen. Juxta-epithelial areas were highly
cellular, showing fibroblasts with a foamy nucleoplasm and inflammatory cells. H. & E.
x214.

FIG. 9. Section of the buccal mucosa painted for 8 months showing cell dissociation, atypical

nuclear staining and invagination of epithelial cells into the corium. There is a chronic
inflammatory exudate and much fibroblastic proliferation in the corium. H. & E. x 107.
FIG. 10.-Section from the buccal mucosa of DOCA treated animal painted for 1 month.

Picture shows an area of marked hyperplasia, and a prominent stratum granulosum. There
are marked acanthotic epithelial cords projecting into the corium, individual cells showing
dissociation dyskeratosis and hyperchromatic nuclei. H. & E. x 214.

FIG. 11.-Section of the buccal mucosa of DOCA pretreated animal for 5 months. There is

hyperplasia and a very prominent stratum granulosum. Invagination of the rete pegs into
the corium is marked. The subepithelial mucosa shows oedema and much fibroblastic
proliferation. H. & E. x 65.

FIG. 12.-Section of the palate of DOCA treated animal painted for 5 months. Picture shows

pale to intense uneven staining collagen with chromophobic areas. An occasional fibre is
abnormally stained orange/blue or orange (black in the picture). Mallory's Trichrome. x 90.
FIG. 13.-Section of the buccal mucosa painted for 6 months showing areas of coarse collagen

fibres, stained partially orange blue. Mallory's Trichrome. x 180.

FIG. 14.-Electron micrograph of isolated collagen from palate of DOCA treated 4 months

control animal. Long stranded collagen with a normal 640 A periodicity is seen. There is
mild lateral swelling of the fibrils and increased amorphous material. Cr shadowed.
x 27,500.

FIG. 15.-Electron micrograph of isolated collagen from the palate painted for 2 months. Long

stranded normal collagen with slight lateral swelling and mild increase in amorphous material
are seen. Cr shadowed. x 21,600.

306

BRITISH JOURNAL OF CANCER.

1                                2

??. ,-s?

?

.,?', :s

4

6

Sirsat and Kandarkar.

3

.:s~ .
: z

VOl. XXII, NO. 2.

BRITISH JOURNAL OF CANCER.

v,I,i ...^;.

3CSSen.#. })7

A                                               10

11                                           12

Sirsat and KandaFkar.

8

VOl. XXII, NO. 2.

1

i

%7

v .9

BRITISH JOURNAL OF CANCER.

13                                   14

15

Sirsat and Kandarkar.

VOl. XXII, NO. 2.

ORAL MUCOSA CHANGES IN THE WISTAR RAT

I P
r. l

1-

01

K -1             III

-     il

0  1x 1

0 V  -1   I   I  I

0  - I I   I I

Iv I I II

Co

0 -  I  I n

0 -  -I   -

Iw 1010<

0vl - - -
1w   10  10   1  10
-{ 0 - - - -

fWI I 110

10 I I I -

0 " 0,a

C;)

1-s

0 *

o  I

CV .

11 11
V S

Co
e0
Co

Ct

Co

4,_

0
*Cob

0
Co
0O

H

r -?

?12
C)0
121 C2C.)
01 ?

?

ii I

I{?I

12 r ?

I C)0
012i C)C)

0

0

L ?

12 r

tII I C)0
01 OC)

0

?     0

? ?

..?

?s r ?

1? I C)0
*?12 I C)C)

1201 ?

? I ?

1 0

?

C -?
I ?12
? I C)0

.? j ?

0
C) -?

?

L ?

? F

0

?     0

?

L

12

?

It         I1CO    Jt

1 0

I            IC        I CD

I_     I~     01I

I el    I v    O

, - _  I     1 a

j -     4  --   J C O
I 4    -4     I z1-

I   -    1- _   .I

- CC

- o
- o

-

I ~   --         I --

I 1            0 1    I   0 1

^. I         cq        c
cq          cs        a
e.          ce        C

CO0    - -

0l1    C01

- O0

- 00

O

O

eD

co   )      100 C    10C~ o

co             C c   O  0 1

(I.         ce       c

0~~~~~~

-~ ~~~~~~~~~~~~~~~~~-

4a o E               ) %

?    H,  r1           C1

28

307

0

0

I-

0

E

(1)
s

.)  .

*eQ

0)

p)

CO

Co

Co
C.)

EH

-4 -4

1.

SATYAVATI M. SIRSAT AND S. V. KANDARKAR

CC                 I     C>
:   IC ?  IC]  IC1  C]

00       m~~~~~

C)                 0~~~~~~~~~~~~~~~~c

"e I~ iX  1 ?  I   1C?

G  e

e                 a   Y~~~~~~~~~~~~~~~e

Ca ~ ~ ~ * ~ C

"l o          -o   CeC s     t

0  x   0~~~~~C~.4

I                        0C  ) .

* cC

C)~~~~~~C              CCb

C

C)

CC   t     CC)"  CC

-         X~~~~~~E-
S   I           C)h.

_     O a~~~~~~~~.

o+~

C                        0

C   sOO. s C O .  CO  COCO

,.5       C]   CC    C    0

. - . ** w . * . * - *

e   e  t   e 4)  4)

<,, ~. E cGt

;~~ ~~~ t _:S w__g.
$,, ~ Hs. H   i

,:                     0

~~ *  ~ ~ C)~4

o~~~~~~~~~~~~~~~~~~~~~~~~~~~~~I

I C)~~~~~~~~~E

CC

C)
C

C.)

Is S

pg

I) -_

Cs

0

-c. 0

oZ

I r  I b  I t  I c

al  Iq       all C ,

t-    I C O      CC CCN

.]     I C]    1 C]     C1i

I t-   I c      I m   >

c   ,   q   I       CN

CZ

I o     I c    I 0   I o   I "

.0

.>          CO ]  O
t* c

IsC

E

IC)
1.0

N:-

I C9 I (m I = - x

to o  COt  Co- Lo

-C   C1   -

rC

G  I to I H I   Ik
an ) ICC
- 4

ceCC  -

!  CZ ~ C ~ C   C

KO     C> t   -
_. 0n

C)

O e

O -C

CC -

C)  1 )4

C)'=

0

CV r

C
'o c

_S

O2 '

._
, ec0 C)  COO C0O
CD  C]  CC   C]

C  C)

o~ o~ o       t

.   *  .  *  *  .C)CC

.5  '5-    C) C)t

~~~~~~ ._

.S     *   '?tDCC

%    -":DI

5X    C.   .

- -zE         $

308

C)

C)
C)
C2)

C1)
C.
C)
C)
0

I-

Ca
C)
C)
:z
9

r C)

2

C)

C)
C)

(t)

z

P l

Ca)
C.)
C)

Ci)
C2)
m

m
C)

It

z

0

C.-

Q

0

0

Ca

C)

C)

._

S

4-)

C3

Ca
9

Ca

H
0

Ca

-3

I

C)

o _
C)     -
ce     t

-4)
~0

C)
Ca
-."

0

1.0

P.>

CCC

0

C)

PA
C)

*C.)
C)

*00y

C *)

C.)

0

H i;

EC

L

ORAL MUCOSA CHANGES IN THE WISTAR RAT

o V

'0     I;

Ie 0

0o   V

V

0

01=

C)

C?      -

"? 0

0

I                      I

I                      I

I                         I

101

I   I

O C
0 1  0 1

0

C)

01

O

4a ~ ~ ~ ~~ C

.     .

.@ t _

C i *   'z>  * < Z

0-   H    H

Co
Co

GO

-

0a

0

,0

CoO

COo

C.)         C

a

0           r

?3

0

0

0 ;

* CIb

H)

C) ;

0

E) 0

C,

4-)

0

Q 0

0   V

0

0X o

0

0>l
0e  W

3 ')j

I        -          --               I

Cn           I          I              I

I          _          i              I

10      C0
rI"

0

0I

CO 1  -_

jI I i

I t- to

I I -I

0

0

CA)

4 i        A

oa

2 0b e

0      r    -      I

cz)  4-D  4')  4a

Z     g  3     t

309

Co
Co

Co

? C

Co
o

*e

Eq

I                      I
I                      I

I                         I

SATYAVATI M. SIRSAT AND S. V. KANDARKAR

In the experimental animals, the distribution of mast cell in the palate was very
sparse, while in the buccal mucosa they were very few to moderate to considerably
increased in number in the four groups.

Submicroscopically, untreated control animals in all four groups showed normal
long-stranded collagen with a 640 A periodicity (Fig. 14). The only submicro-
scopic alteration seen in the deficient and DOCA-treated experimental animals
was a mild lateral swelling. The DOCA-treated group also showed some increase
in amorphous material. There was no loss of the 640 A periodicity (Fig. 15)
which was not often obscured by a coating of increased amorphous material.

DISCUSSION

This study should optimally have been carried out on a number of species
simultaneously. However, designs of experiment are often limited by technical
difficulties. It was possible therefore to test only one rodent species, the Wistar
rat, which has been maintained in a controlled inbred state since its import in
1942. Varied experimental conditions have been obtained by inducing dietary
or hormonal stress in parallel with exposure to the irritant.

The role of diet in the causation of changes in the oral cavity has been men-
tioned (Sharp, 1956; Wolbach and Bessey, 1942; Forbus, 1952; Martin and Koop,
1942; Khanolkar, 1944; Shanta and Krishnamurthi, 1963). The lack of protein
in the diet produces many tissue changes in the oral cavity such as thinning of the
mucosal epithelium (Stahl, Sandler and Cahn, 1955; Unakar, 1960) and retarded
wound healing (Udupa, Woessner and Dunphy, 1956). Vitamin B has been
correlated with oral mucosal changes by a number of investigators. Martin and
Koop (1942) have implicated vitamin B deficiency in the etiology of degenerative
changes in the human oral mucosa usually antedating malignant transformation.
In this investigation therefore two groups of WVistar rats consisting of 35 and 36
animals were deprived of protein and vitamin B-complex respectively. The
animals kept on a B-complex deficient diet were not given an additional dose of
any sulpha compound as the aim was to induce only a mild deficiency such as that
found in malnourished Indian people and not a total lack of the vitamin.

The use of DOCA seems to need some explanation. Reports describe an
enhanced tissue reaction with the use of certain anabolic hormones (Bauer and
Clark, 1953; Pirani et al., 1950; 1951; Sirsat and Khanolkar, 1960; Ketkar and
Sirsat, 1966). There was originally some confusion as to whether DOCA was
responsible for the production of inflammation per se. A detailed study was
carried out by Ketkar and Sirsat (1966) on the exact morphological and histo-
chemical changes in connective tissue treated with DOCA. They concluded that
DOCA itself did not induce inflammation. When the trauma of injection accom-
panied DOCA administration the inflammation was more marked than that in
which injection injury was inflicted without parallel hormone administration. In
a study of capsaicin treatment in DOCA pretreated oral mucosa in the Wistar
rat, Sirsat and Khanolkar (1960) reported a total increase in sensitivity to cap-
saicin, observed optically and submicroscopically, in the hormone treated animals.
It was in the light of these observations that this anabolic mineralocorticoid was
used in this study.

The histology of the untreated control palate, carried out in these studies, also
notes the marked divergence in the number of epithelial cell layers which normally

310

ORAL MUCOSA CHANGES IN THE WISTAR RAT

occur in different parts of the palate (Kutuzov and Sicher, 1952). It was therefore
extremely necessary, that in a study which takes into account cell multiplication.
care should be taken and uniformity maintained in the palatal area being described.
This study compares mainly the palatal epithelium of the rugal ridges, in the three
groups-normal untreated, solvent control and palates painted with the lime.
This however did not rule out description of marked change occurring in other
parts of the palate.

It is seen throughout that the buccal mucosa presents a greater degree of tissue
clhanige than that observed in the palate. Observations on control and treated
oral mucosa described in this paper lead one to conclude that in the Wistar rat,
hyperplasia alone should not be considered a specific pathological change pro-
voked by the irritant. It appears to be more a biological disturbance seen
routinely in the rat palate as a constant reaction to mastication of solid food.
Invagination of rete cones into the papillary layer was found in 59' lime-treated
animals, while frank acanthosis occurred in only 28. Similar acanthosis has
been noted by Dunham. Muir and Hamner (1966) in 26 hamsters treated with
calcium hydroxide. Two valid conclusions are possible from this study of the
oral epithelium- (1) In animals not exposed to an extraneous traumatic agent,
continuous injury due to solid diet, coupled with enhanced sensitivity of the
mucosa due to dietary or hormonal stress can occasionally produce an epithelial
change which can be called abnormal, (2) Acanthosis of the oral epithelium is
found only in animals exposed to lime, and reflects probably a deeper injury to
the cellular growth pattern and also a chronically altered epithelium connective
tissue interrelationship.

Table III shows the inflammatory response in the control and experimental
ainimals. The palate and buccal mucosa of all experimental animals react to the
lime-inflicted trauma with marked dilatation of blood vessels and inflammatory
exudation. Inflammation in response to the whole betel chew has been men-
tioned by Muir and Kirk (1960). These authors painted the ears of 53 Swiss
mice with an aqueous extract of a typical Singapore tobacco, betel nut, shell
lime " quid " for 2 years and produced a chronic inflammatory exudate with a
rather oedematous dermis after 5 months of painting. In this study, the presence
of protein and vitamin B deficiency and the exposure to DOCA no doubt were
factors that augmented the oral tissue response to this highly traumatic in-
gredient of the betel chew.

Like the epithelial reaction, the mast cell response appears to be considerably
more marked in the buccal mucosa than the palate. This could really be so, for
the same reason suggested for the greater epithelial hyperplasia in the buccal
mucosa than in the palate-a more intimate exposure to the irritant. A point
h-as however to be kept in mind that even in untreated controls, under normal
conditions, the palatal mast cell population is much lower than in the buccal
mucosa. Even so, proportionately speaking, the mast cell response is very marked
in the buccal mucosa in all four groups and suggests again the highly irritant
nature of lime and the great susceptibility of the subepithelial tissue to this
substance.

Subepithelial hyalinisation was a marked feature in the oral mucosa of all
animals exposed to lime. The biological significance of this phenomenon, which
is a non-specific limited connective tissue response to injury, and the epithelial
reaction provoked by altered connective tissues have often been mentioned

311

SATYAVATI M. SIRSAT AND S. V. KANDARKAR

(Gillman et al., 1955; Sirsat and Pindborg, 1967). Suffice it here to emphasise
that the epithelium overlying a hyalinised submucosa is an uneasy one, mainly
due to a defective nutritional status resulting from the replacement of an active
vascular tissue by an inert hyalinised substance. The abnormal staining reaction
seen in the palates of 70 animals and in the buccal mucosa of 99 out of 115 animals
exposed to lime fall within the definition given by Gillman et al. (1954, 1955) for
a pseudoelastic transformation of the collagen fibres. Submicroscopically,
however, the only change seen in the collagen fibrils isolated from the experi-
mental animals is a lateral swelling. No frank alteration to degraded material
similar to that seen in human oral submucous fibrosis (Sirsat and Khanolkar,
1957) and rat mucosa treated with capsaicin (Sirsat and Khanolkar, 1960) is
found. Therefore the tinctorial changes do not reflect an outright transformation
to frankly pathological degradation of an elastotic nature. Recent work on atypi-
cal staining of collagen suggests another possibility. Scheuner and Gabler (1965)
carried out a comparative histological and histochemical study of collagen and
elastic fibres in sections from 28 human organs and tissues froin newborn to 28
years old, taken 0-7 days post-mortem, and in dogs and rabbits. Staining was
done with a wide spectrum of 13 compounds. The factors involved in the aberrant
staining of collagen were not ageing, post-mortem or pathological effects. These
authors admit a similarity between the atypical stained collagen and fibrinoid in
degraded connective tissue, but conclude that this phenomenon occurs not
uncommonly in non-pathological collagen also. Craik and McNeil (1966) studied
the differential staining of human collagen from the abdominal wall, after subject-
ing it to stress by artificial stretching. The fibrous reaction to stress was tinctorial
alteration to red in Mallory's trichrome. They felt that the stretching probably
reorientated the collagen structure, removed the aniline blue positive mucopoly-
saccharide and exposed fuchsinophilic protocollagen molecules. Melcher (1963)
also described such altered staining of collagen in gingival inflammation.

In this investigation, too, much tissue damage is inflicted with the presence of
oedema and continuous inflammation and a number of stressor agents come into
play. The change in the collagen is seen only at the optical levels, tinctorially so,
there being no submicroscopic degradation in fibril structure.  The altered
staining in this study could therefore also be due to a reorientation in the physical
properties of the fibrils due to stress and not to any pathological reorientation at
molecular levels in the basic structure of the scleroprotein fibril. Lateral swelling
in the fibrils found submicroscopically confirms the swelling seen optically in all
lime treated collagen. This swelling is brought about by an overall tissue hydra-
tion due to the pathological oedema and is a non-specific phenomenon. At the
level of the fibril structure it reflects a change in the inter-molecular bonds with
increased width of the chain.

The indirect aim in animal studies such as this one is always to evaluate the
test substance as a possible causative agent of human oral disturbance. The
correlation is attempted by a comparison of tissue changes in the human dyscrasia
in question and the experimental study. Three main oral disorders come to
mind in connection with the oral mucosa in the Indian people-submucous
fibrosis, leukoplakia and frank oral cancer. In view of the tissue changes pro-
duced by commercial lime in the rat oral mucosa, it is worth discussing its pro-
bable role in the etiology of morbid conditions of the human mouth. Submucous
fibrosis of the oral mucosa has been recognised as a not uncommon entity found

312

ORAL MUCOSA CHANGES IN THE WISTAR RAT

mainly in the Indian population (Joshi, 1953; Pindborg, 1965; Sirsat and Khanol-
kar, 1957, 1960) and occasionally in other south-east Asia countries (Pindborg
and Sirsat, 1966). The main tissue reaction in this condition is epithelial atrophy
in most cases (Pindborg et al. 1965) hyperplastic epithelium being seen in only a
few advanced cases. There is dense hyalinisation of connective tissue, coexistent
with a chronic inflammation (Sirsat and Khanolkar, 1957). Acute inflammatory
changes followed by hyalinisation are found in this study in the lime treated
mucosa also. The epithelial reaction is always a hyperplasia, very different from
that usually seen in submucous fibrosis. It is very unlikely that the acute injury
inflicted by lime could lead to the chronic productive response seen in submucous
fibrosis (Sirsat and Khanolkar, 1960). A clinical reason to support this contention
is that submucous fibrosis can occur in young subjects who have never chewed
pan, and so never been exposed to long-term irritation by lime.

It has already been mentioned that lime has been correlated many years ago
to the causation of leukoplakia (Fells, 1908; Orr, 1933; Bentall, 1908; Balendra,
1949). More recently, detailed surveys have been reported by Atkinson et
al. (1964) and Forlen et al. (1965). Atkinson et al. studying the oral mucosa of
islanders in New Guinea mention their custom of trailing the mucosal surface with
a stick dipped in shell lime, subsequent to chewing the betel nut. They felt that
the presence of white leukoplakic patches traced the pathway along which the lime
must be continually deposited. Forlen et al. (1965) have reported a correlation
between the use of lime and a high incidence of leukoplakia in these same people.
Leukoplakia in all these studies rightly denotes just the clinical presence of white
patches. The histological aspect of this term postulates the presence of hyper-
plasia, hyper- or para-keratosis, acanthosis and dyskeratosis (Shafer, Hine and
Levy, 1964). In the majority of animals exposed to lime in this study, these
changes were seen in the epithelium. The mucosal epithelium of the rat is
normally more keratinised than that of man (Provenza, 1964); it is also notoriously
more resistant to change induced by external agents. It therefore appears
logical that if lime can evoke a disturbed growth pattern in the rat mucosa, the
human buccal mucosal epithelium would be even more traumatised. Lasting
mucosal changes could then occur. The question that followed concerns the
further involvement of commercial lime in frank oral cancer. In this study,
painting for periods of time up to 12 months did not produce a single recognisable
malignancy, although atypia was seen in some epithelia. This need not rule out
the involvement of this high-grade irritant in the causation of human oral cancer.
On the basis of the moderate to massive epithelial and subepithelial tissue damage,
caused by this irritant, two surmises can be made on the mode by which a long-
term use of the betel chew might lead to oral cancer. A continuous coexistence of
an highly altered epithelium and underlying connective tissue and a chronic
interference with normal metabolic functions could lead to a neoplastic alteration
of the epithelium. The other hypothesis is based on the combined use of stone or
shell lime and tobacco, a practice prevalent in all parts of India, though not so in
New Guinea according to Atkinson et al. (1964). It could be that the tobacco
would exert a surer carcinogenic effect on tissue damaged severely or already
rendered hyperplastic by the lime. Lime could then, when used along with tobac-
co, be termed rather loosely a co-carcinogen. Muir and Kirk (1960) produced
squamous carcinoma or papilloma in the ears of Swiss mice painted daily for two
years with a crude aqueous extract of the whole betel quid. They attributed their

313

314             SATYAVATI M. SIRSAT AND S. V. KANDARKAR

success in obtaining tumours where purer tobacco extracts failed to elicit them
(Mody and Ranadive, 1959) to the possible presence of co-carcinogenic factors in
the whole betel quid. It could well be that lime, which is invariably present in
the betel quid or " pan ", acts thus by virtue of the tissue reaction it provokes, as
the co-carcinogenic agent.

REFERENCES

ATKINSON, L., CHESTER, I. C., SMYTH, F. G. AND TEN SELDAM, R. E. J.-(1964) Cancer

N.Y., 17, 1289.

BALENDRA, W.-(1949) Br. dent. J., 87, 83.

BAUER, W. AND CLARK, W. S.-(1953) Recent Prog. Horm. Res., 8, 217.
BENTALL, W. G.-(1908) Br. med. J., ii, 1428.

COORAY, G. H.-(1944) Indian J. med. Res., 32, 71.

CRAIK, J. E. AND MCNEIL, I. R. R.-(1966) Nature, Lond., 209, 931.

DUNHAM, LUCIA J., MuIR, C. S. AND HAMNER III J. E -(1966) Br. J. Cancer, 20, 588.
FELLS, A.-(1908) Br. med. J., i, 1357.

FORBUS, W. D.-(1952) 'Reaction to injury-Pathology for student of Disease'.

Baltimore (Williams & Wilkins Co.). Vol. II.

FORLEN, H. P., HARNSTEIN, 0. AND STUTTGEN, G.-(1965) Arch. klin. exp. Derm., 221,

463.

GILLMAN, T., PENN, J., BRONKS, D. AND ROUX, M.-(1954) Nature, Lond., 174, 789.
GILLMAN, T., PENN, J., BRONKS, D. AND ROUX, M.-(1955) Archs Path., 59, 733.
JOSHI, S. G.-(1953) Indian J. Otolar., 4, 1.

KETKAR, M. B. AND SIRSAT, SATYAVATI, M.-(1966) J. Invest. Derm., 46, 319.
KHANOLKAR, V. R.-(1944) Cancer Res., 4, 313.

KHANOLKAR, V. R.-(1959) Acta Un. int. Cancr., 15, 67.

KUTUZOV, H. AND SICHER, H.-(1952) Anat. Rec., 110, 275.

MARTIN, H. AND Koop, C. E.-(1942) Am. J. Surg., 57, 195.

MELCHER, A. H.-(1963) In 'Structure and function of connective and skeletal tissue'.

Edited by Fitton Jackson, S., Harkness, R. D., Partridge, S. M. and Tristram,
C. R. London and New York (Butterworth) p. 500.

MODY, J. K. AND RANADIVE, K. J.-(1959) Indian J. med. Sci., 13, 1023.
MUIR, C. S.-(1962) Cancer, N.Y., 16, 584.

MUIR, C. S. AND KIRK, R.-(1960) Br. J. Cancer, 14, 596.
ORR, I. M.-(1933) Lancet, ii, 575.

PIRANI, C. L. AND STEPTO, R. C.-(1950) Am. J. Path., 26, 749.

PIRANI, C. L., STEPTO, R. C. AND SUTHERLAND, K.-(1951) J. exp. Med., 93, 217.
PINDORG, J. J.-(1965) Bull. Wld Hlth Org., 32, 748.

PINDBORG, J. J., CHAWALA, T. N., SRIVASTAVA, A. N. AND GUPTA, D.-(1965) Acta

odont. scand., 23, 277.

PINDBORG, J. J. AND SIRSAT, SATYAVATI M.-(1966) Oral Surg., 22, 764.

PROVENZA, V. D.-(1964) ' Oral Histology, Inheritance and Development.' London

(Pitman Medical Publishing Co. Ltd.), Philadelphia (J. B. Lippincott Company)
p. 364.

SCHEUNER, G. AND GABLER, W.-(1965) Acta histochem., 20, 91.-Cited in Collagen

Curr., 6, 251.

SHAFER, W. G., HINE, M. K. AND LEVY, B. M.-(1964) 'A text book of Oral Pathology.'

Second Edition. Philadelphia and London (W. B. Saunders Co.) p. 85.
SHANTA, V. AND KRISHNAMURTHI, S.-(1963) Br. J. Cancer, 17, 8.
SHARP, G. S.-(1956) Acta Un. int. Cancr., 12, 135.

SIRSAT, SATYAVATI M. AND KHANOLKAR, V. R.-(1957) J. Path. Bact., 73, 439.-(1960)

J. Path. Bact., 79, 53.

ORAL MUCOSA CHANGES IN THE WISTAR RAT                   315

SIRSAT, SATYAVATI M. AND PINDBORG, J. J.-(1967) Acta path. microbiol. scand. 70,

161.

STAHL, S. S., SANDLER, H. C. AND CAHN, L.-(1955) Oral Surg., 8, 760.

UDUPA, K. N., WOESSNER, J. F. AND DUNPHY, J. E.-(1956) Surg. Gynec. Obstet., 102,

639.

UNAKAR, N. J.-(1960) ' Biological Studies with the Electron Microscope with reference

to the Mesenchymal Response in Experimental Rat Liver Cirrhosis.' A thesis
submitted to the University of Bombay for the degree of Master of Science.
WOLBACH, S. B. AND BESSEY, 0. A.-(1942) PAys. Rev., 22, 233.

				


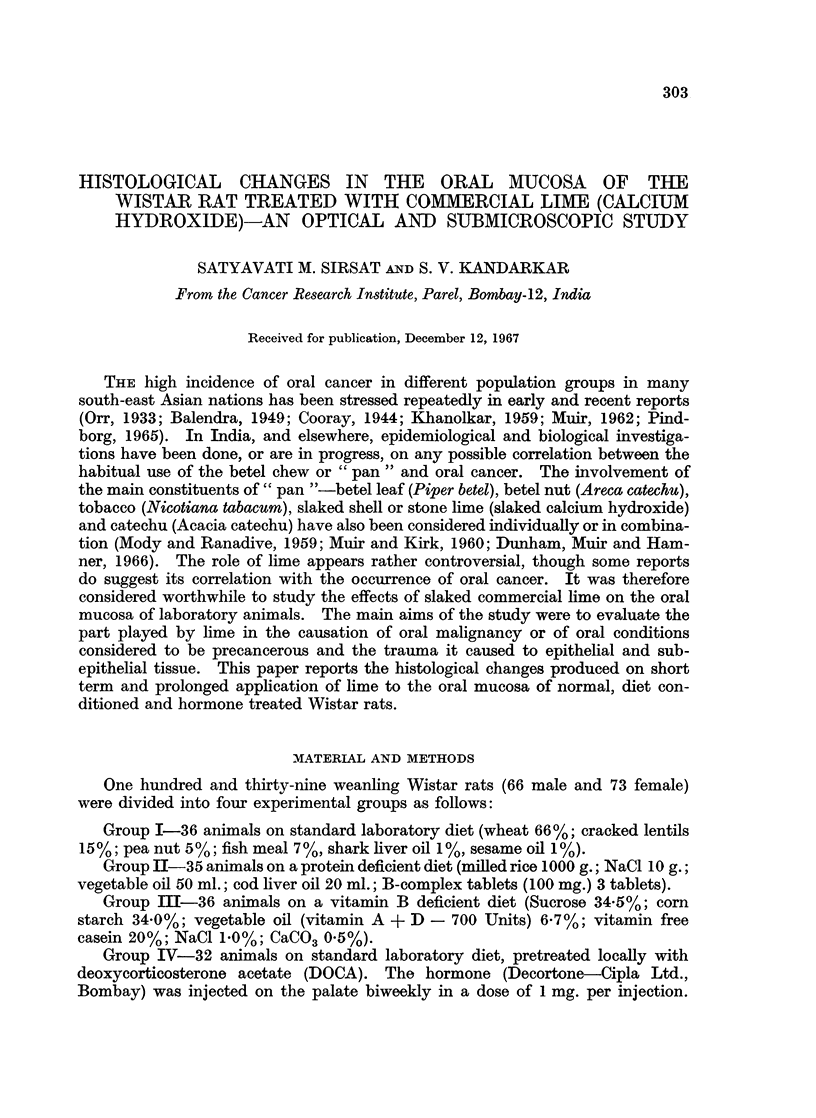

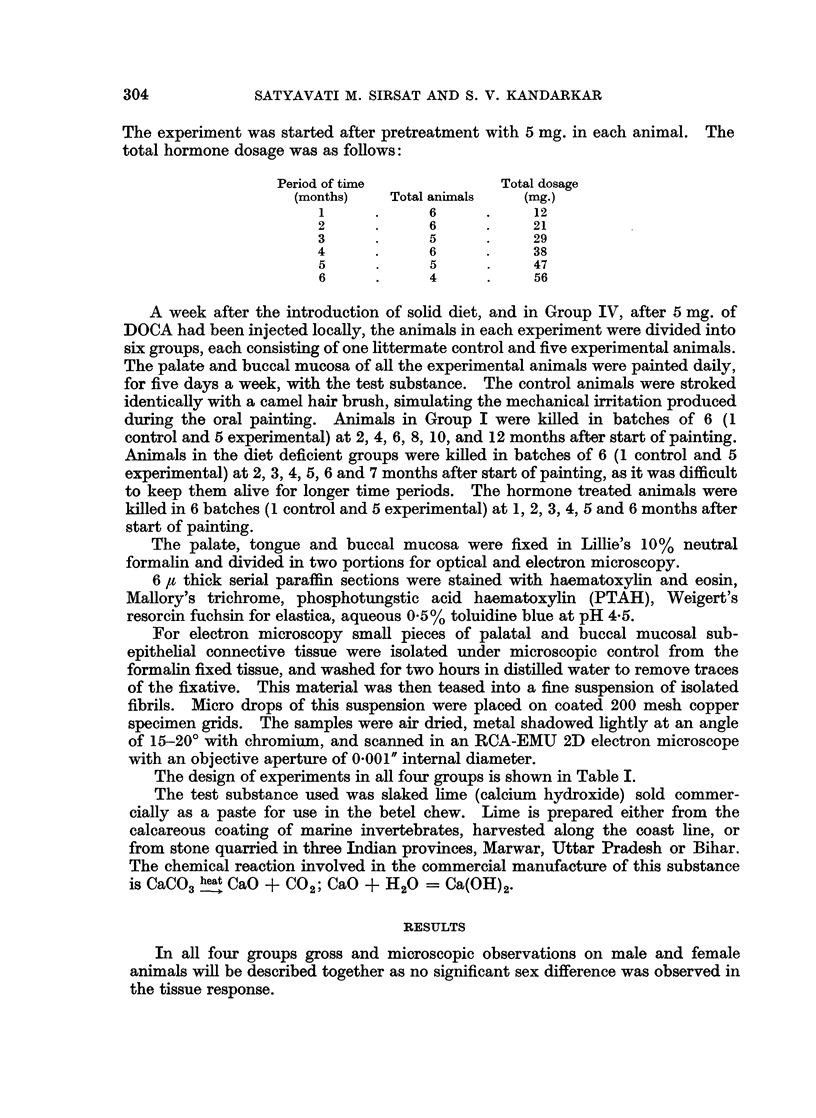

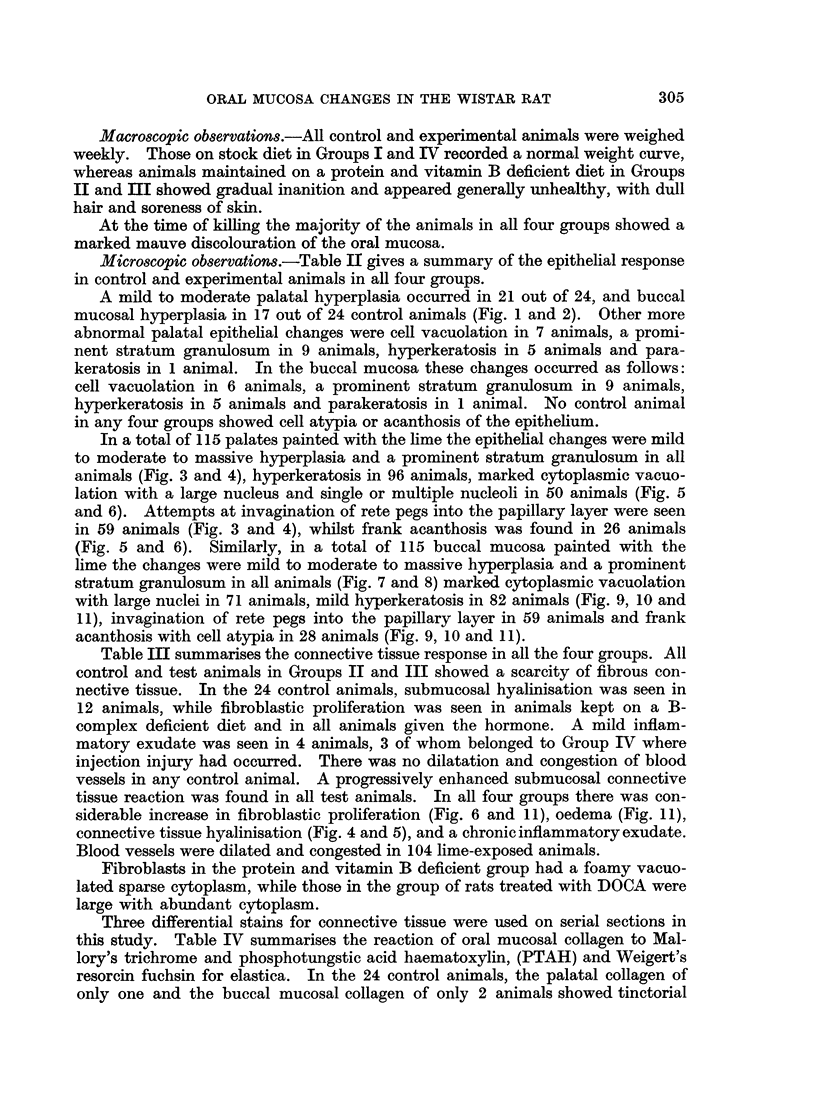

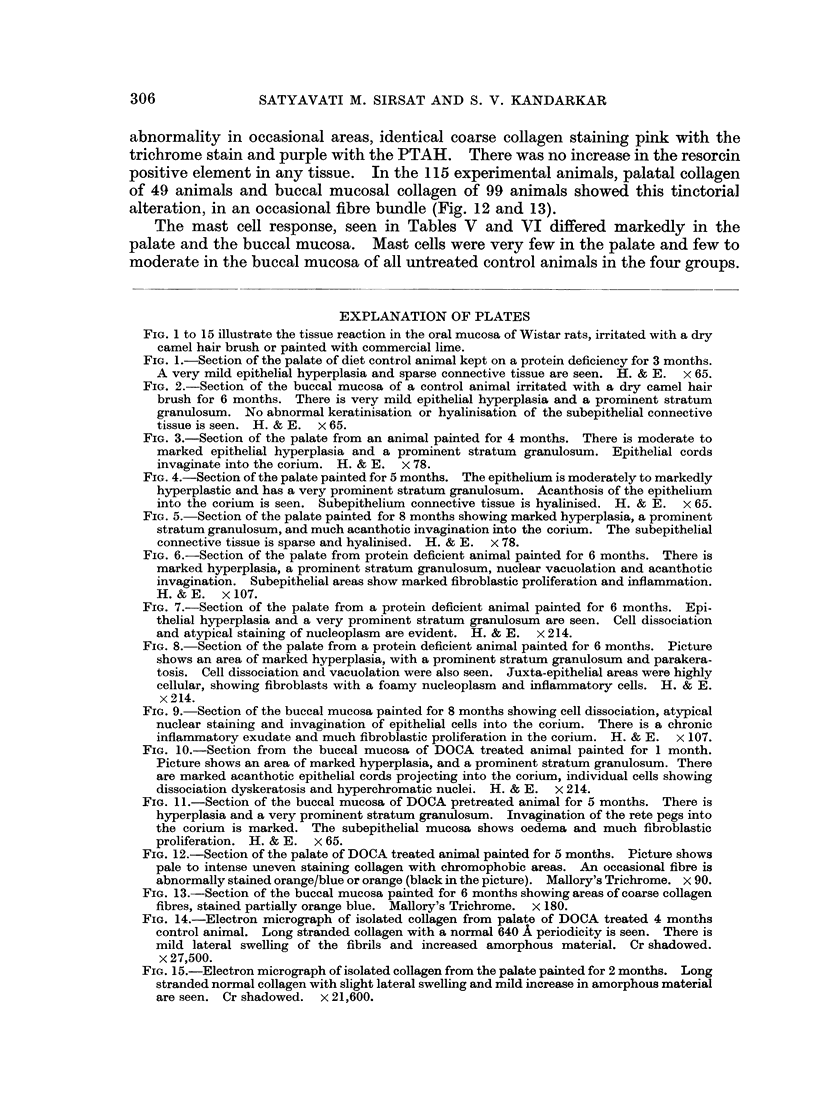

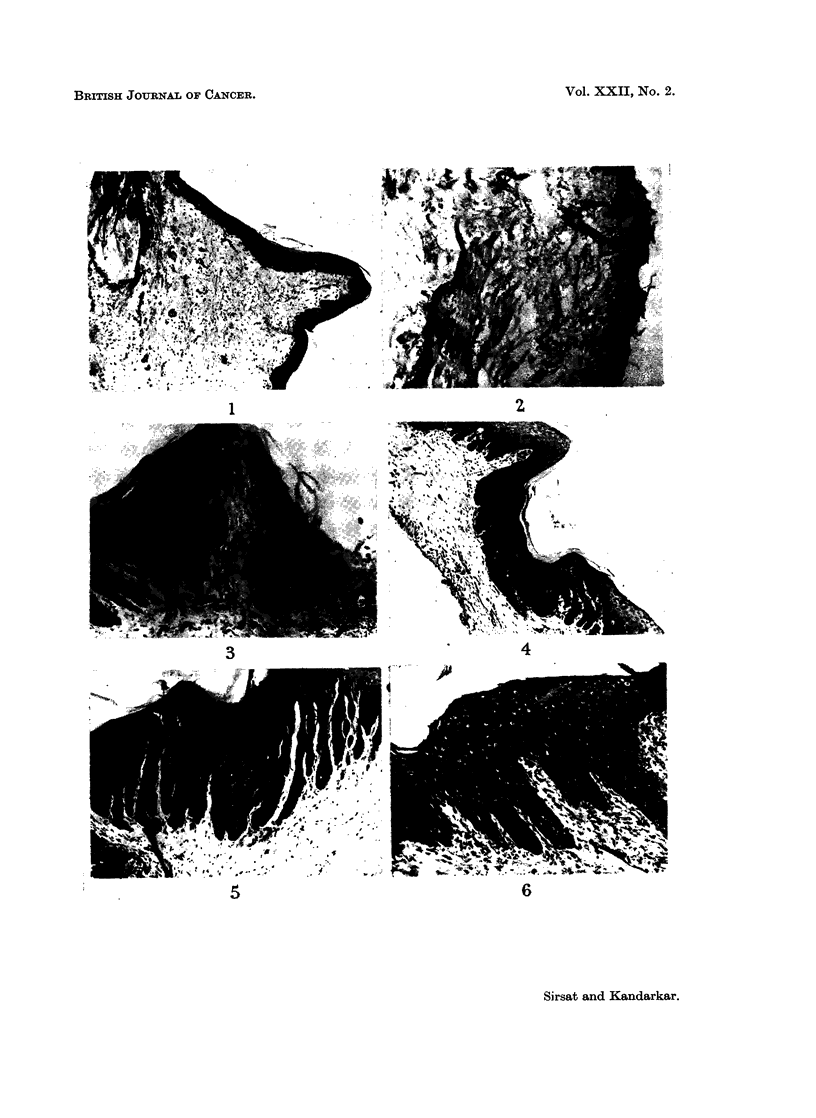

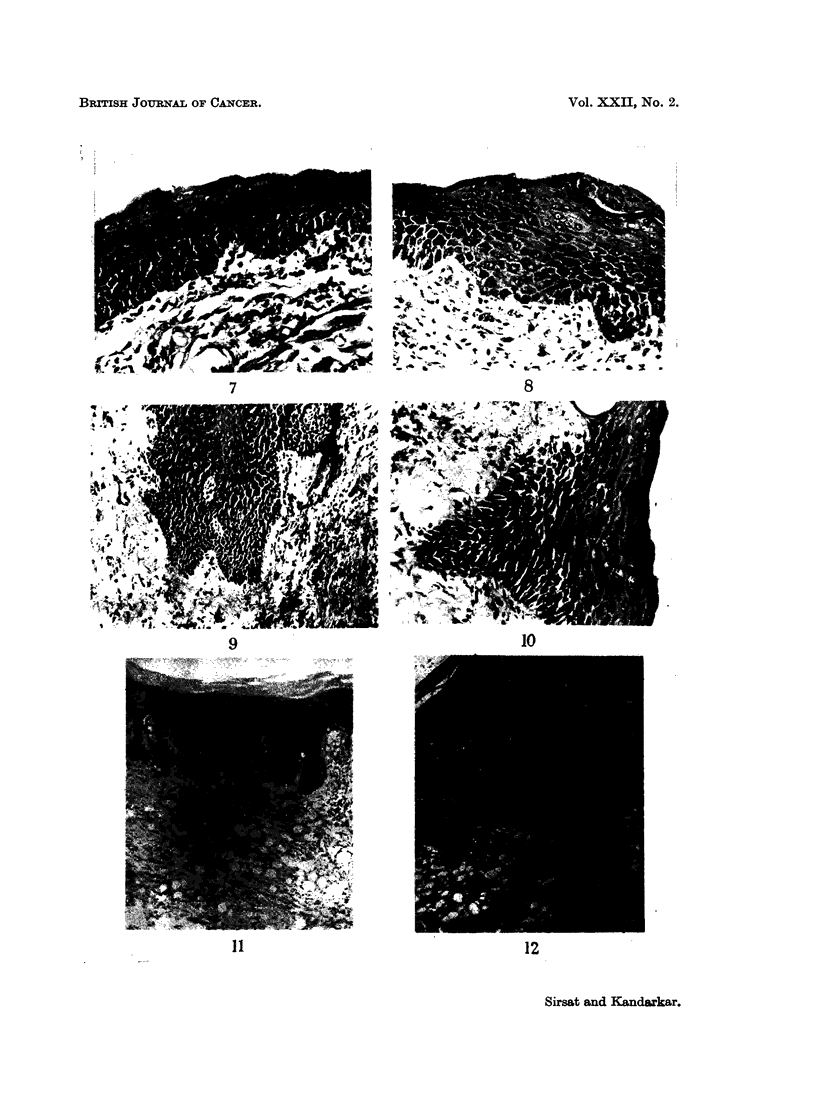

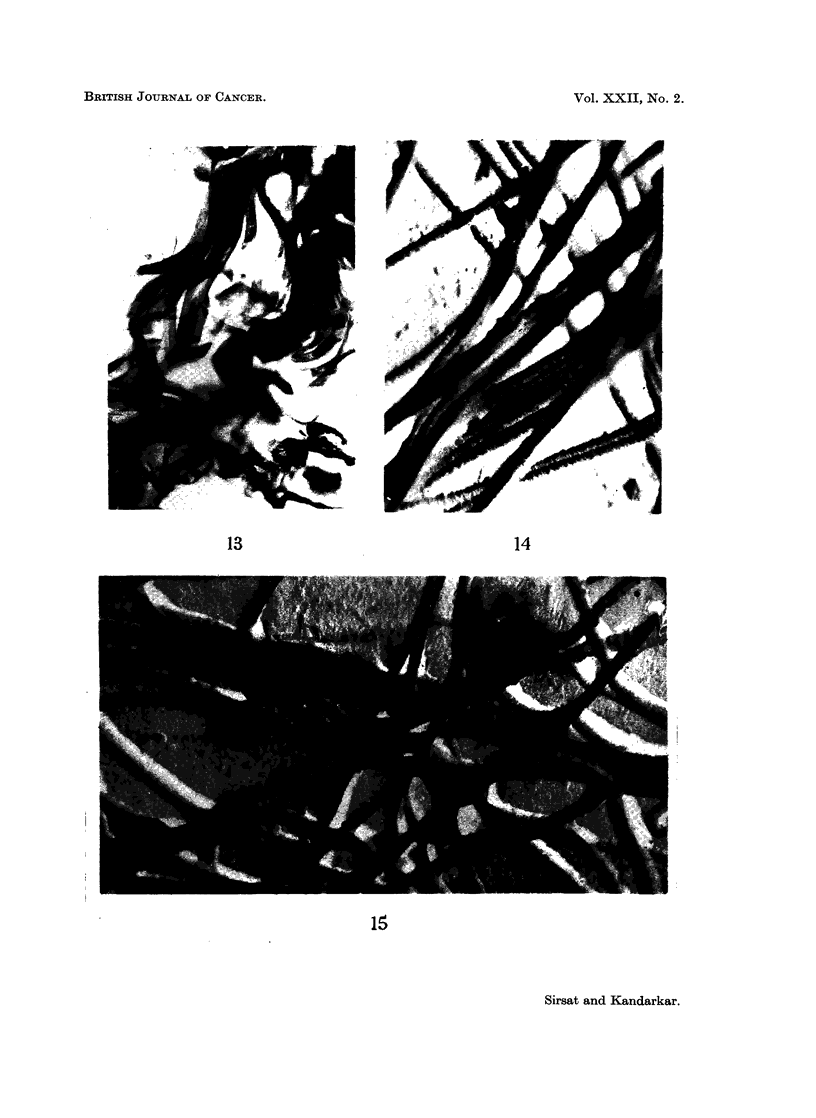

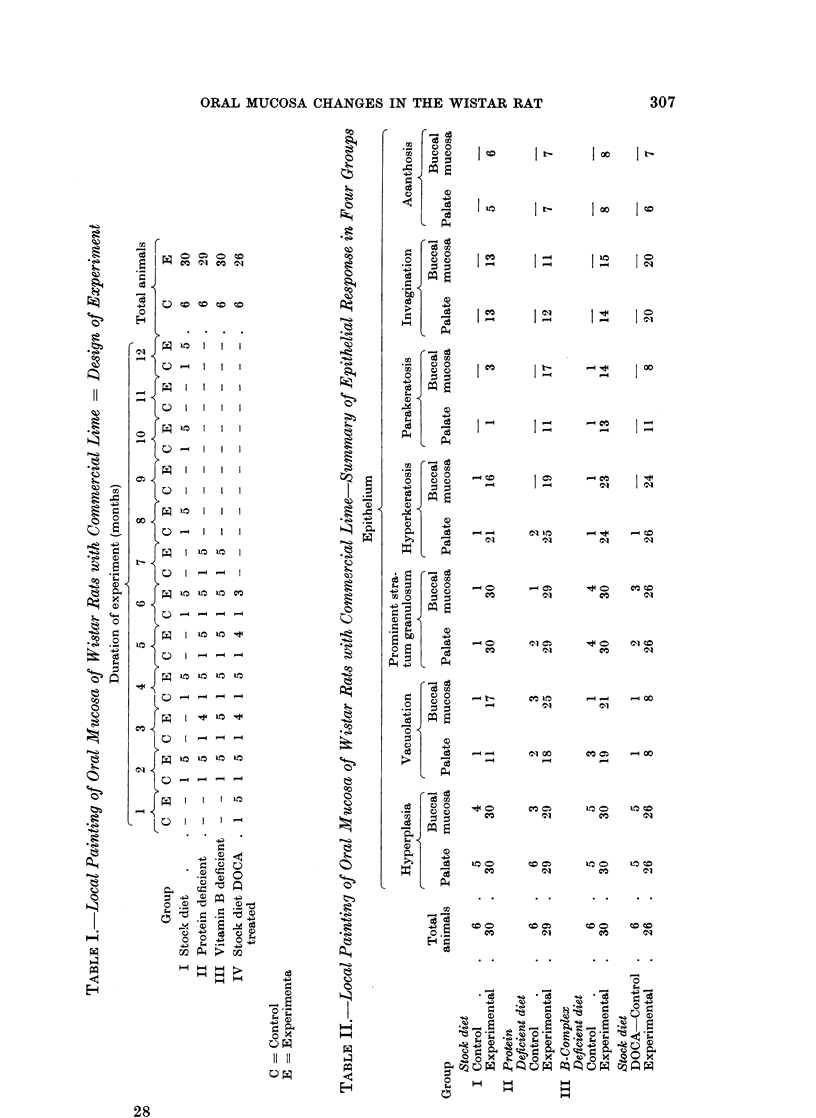

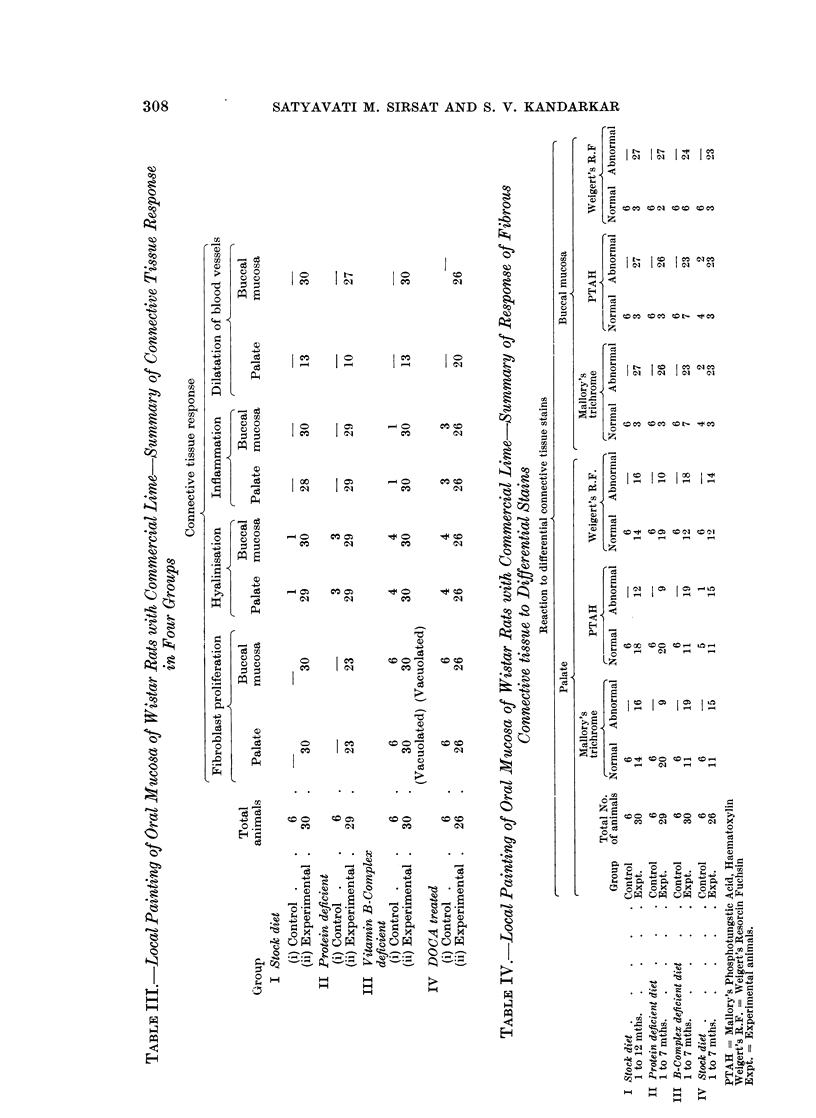

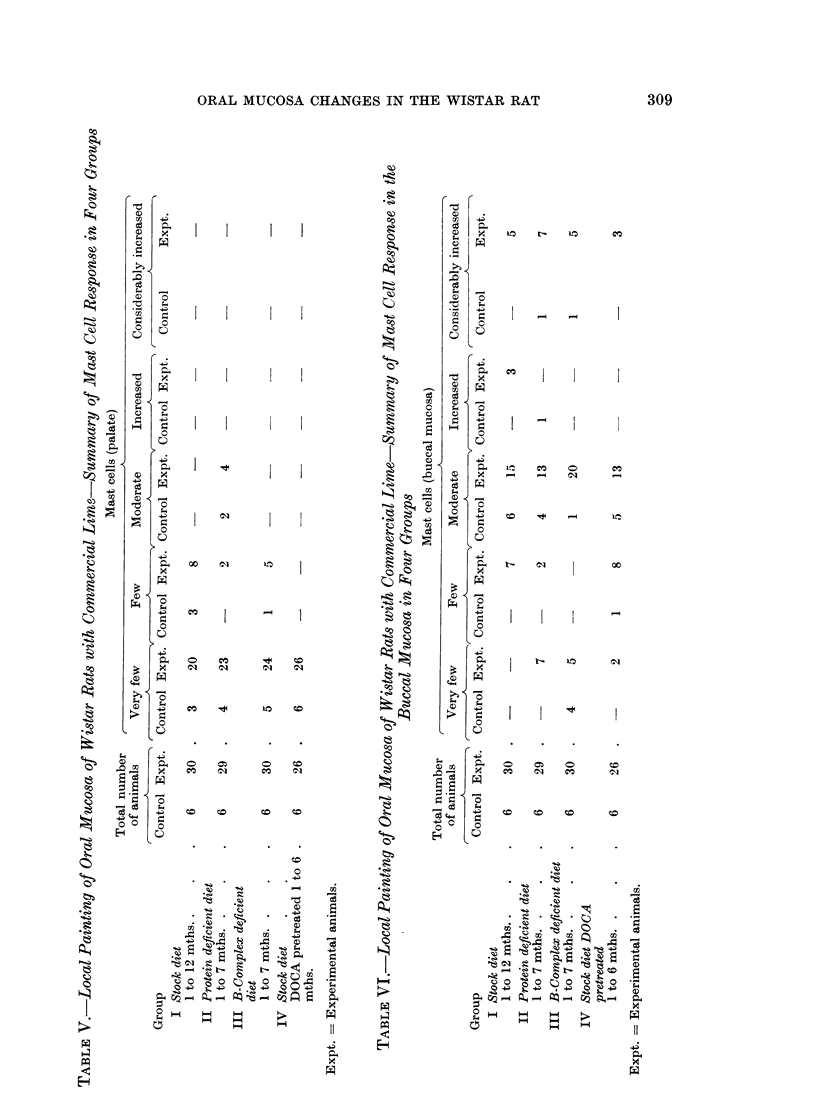

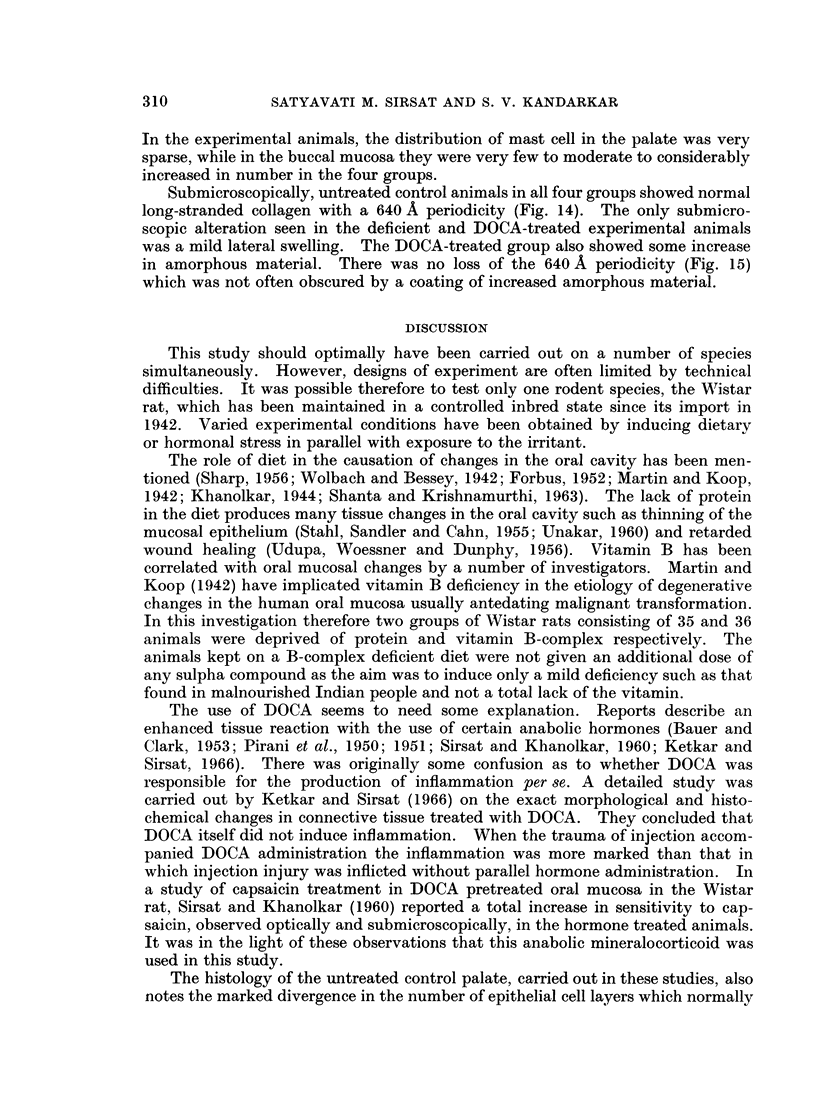

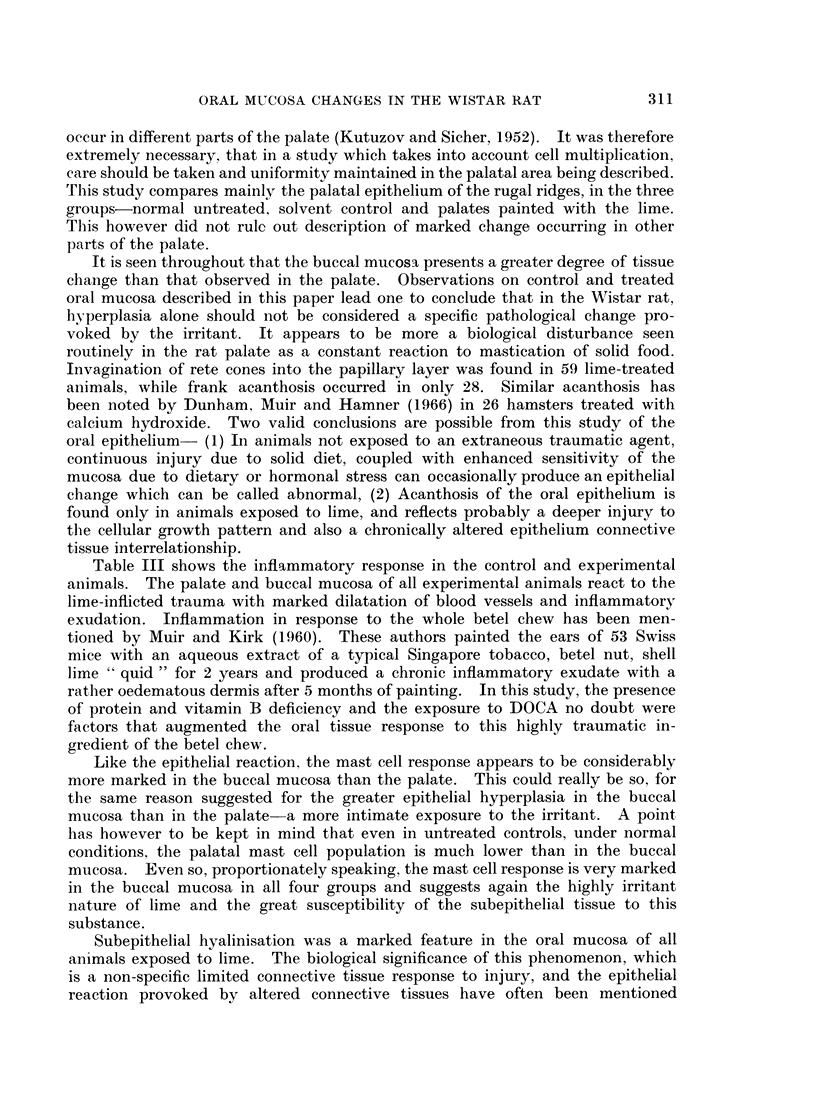

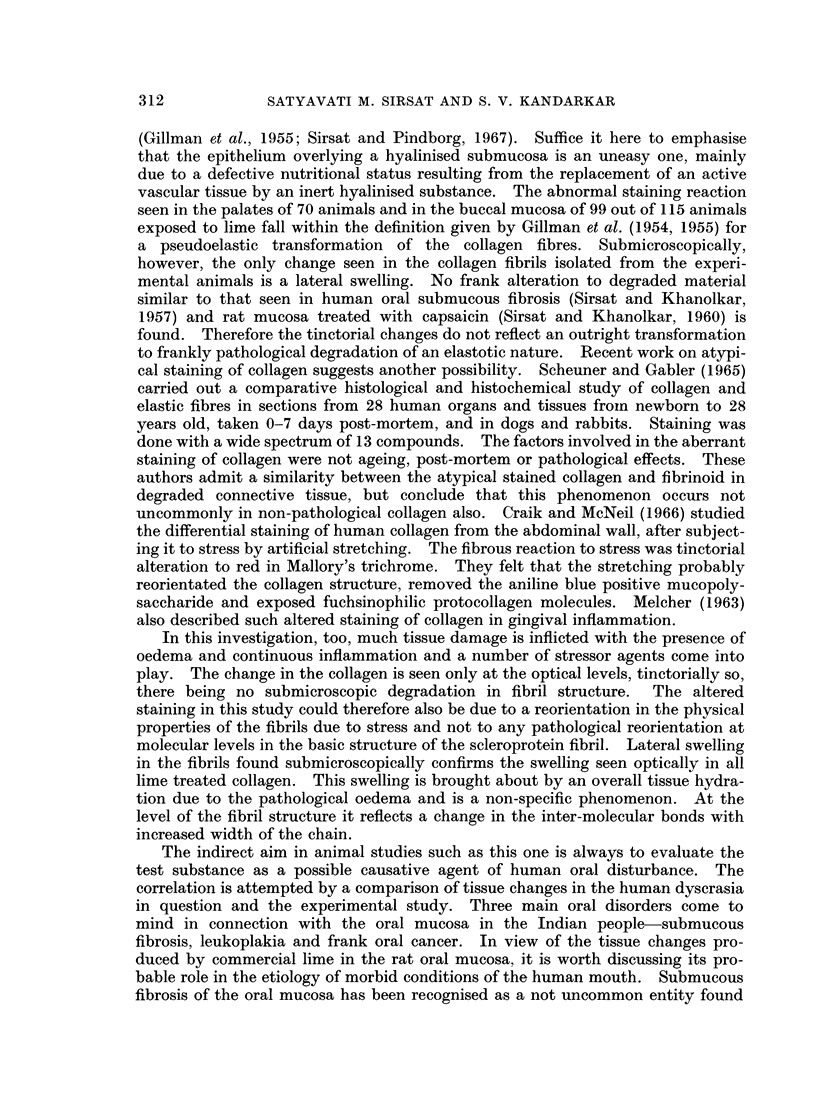

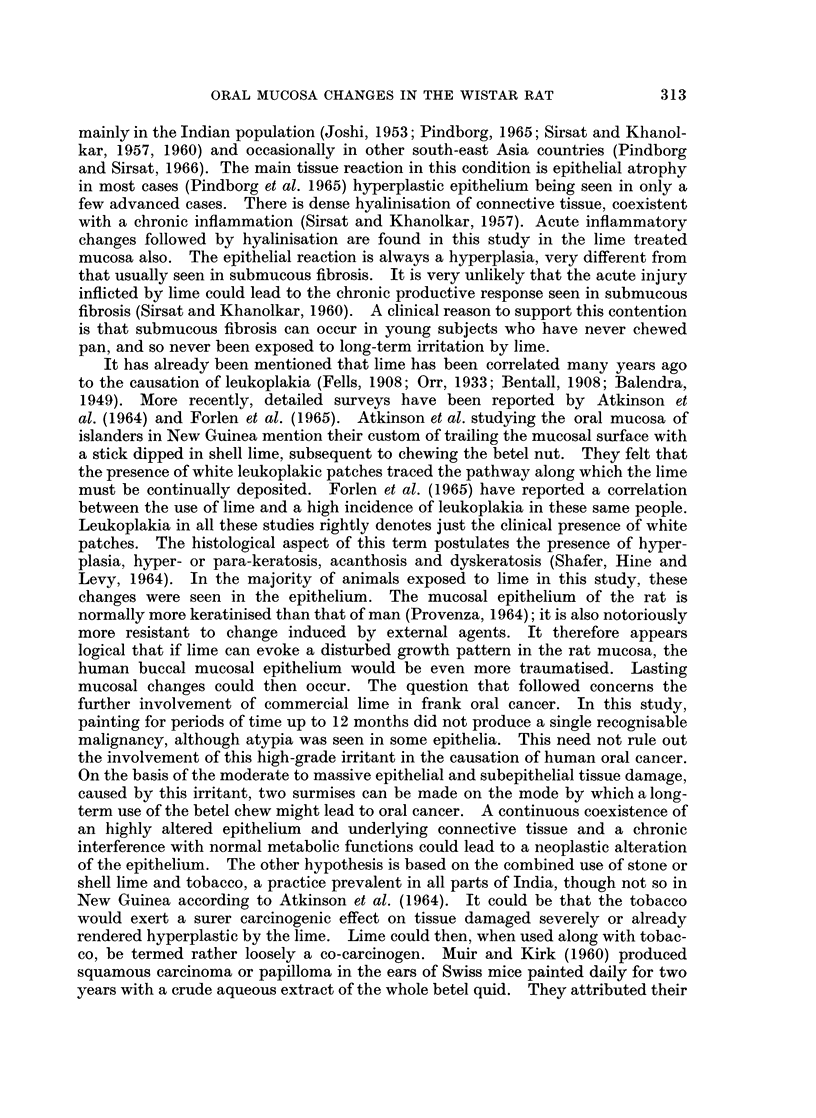

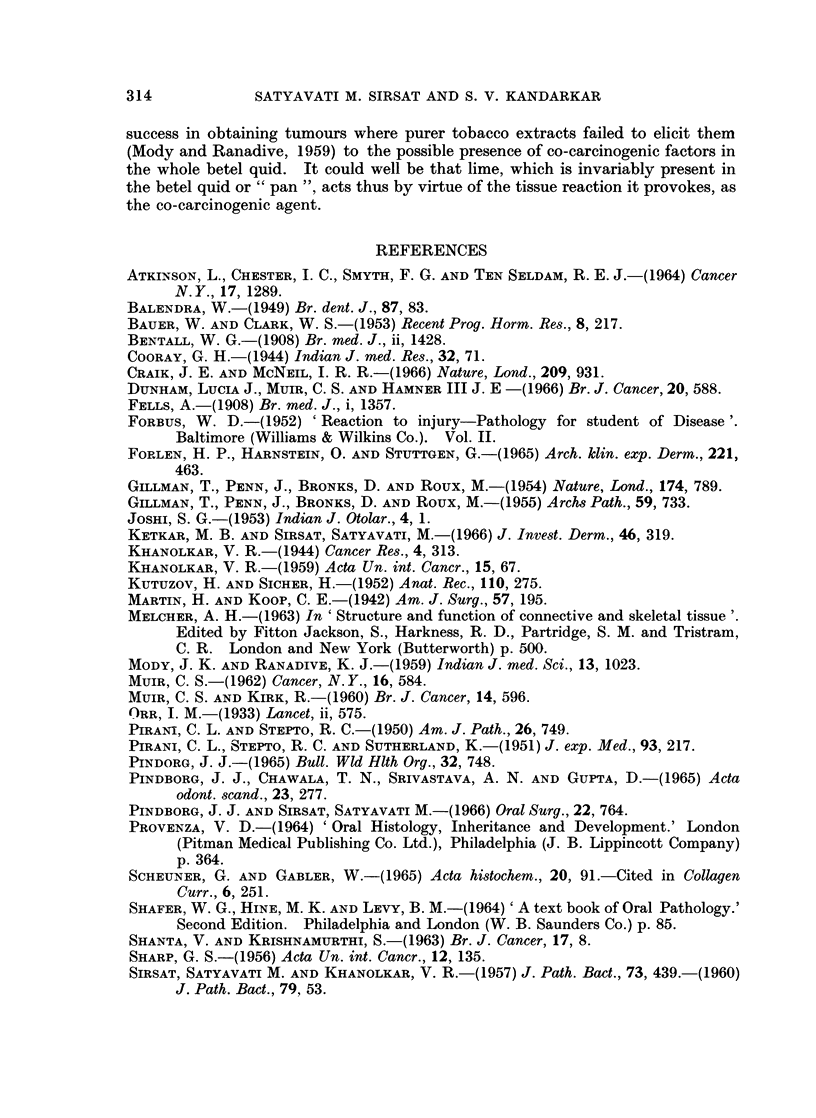

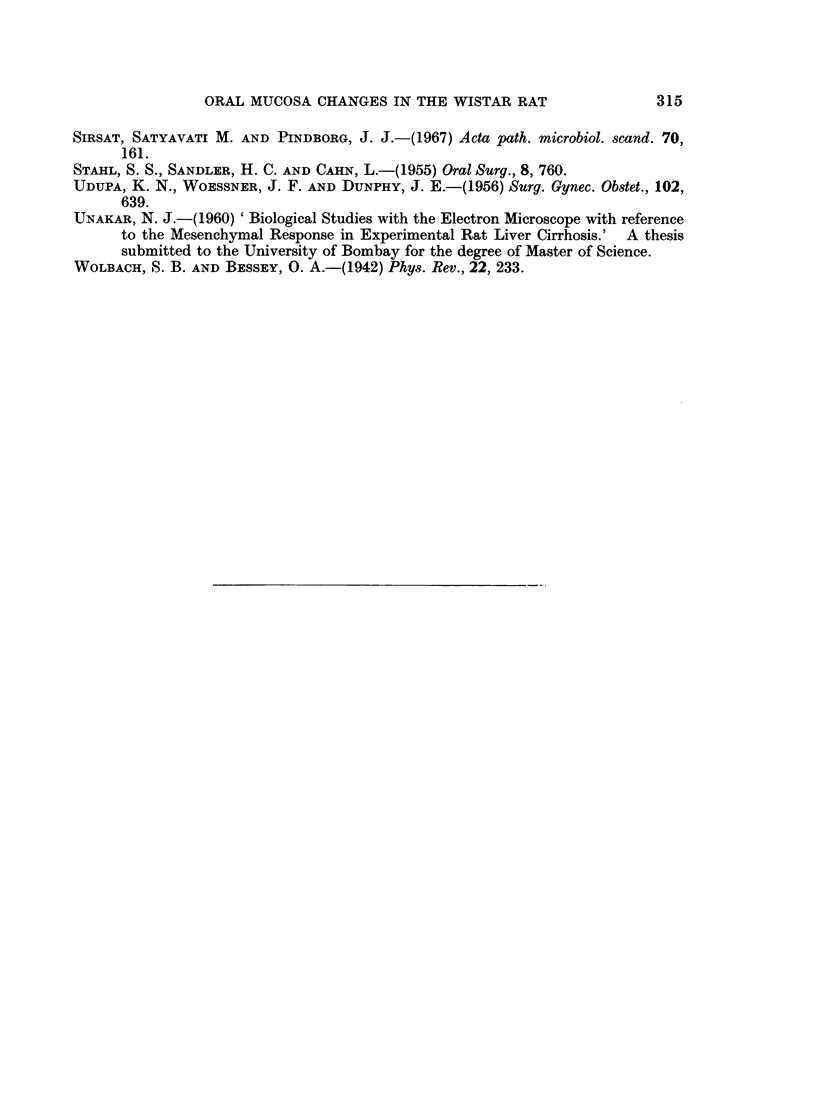

